# RKMR: A Rapid Kernel Machine Regression Framework for Optimal Marker Detection in Spatial Omics Data

**DOI:** 10.64898/2026.07.27.740999

**Published:** 2026-08-03

**Authors:** Souvik Seal, Anirban Chakraborty, Chloe Mattila, Mark Rubinstein, Peggi Angel, Debashis Ghosh, Dongjun Chung, Brian Neelon

## Abstract

High-throughput spatial omics technologies enable molecular profiling within intact tissue architecture, yet identifying concise, predictive, and biologically interpretable marker panels for cell types, tissue domains, and disease-associated tissue classes remains challenging. This limitation hinders the development of actionable panels for targeted validation and downstream translation. Existing pipelines rely largely on univariate differential-expression analyses, which ignore joint molecular structure and provide limited predictive insight. Multivariate machine-learning methods, including random forest, XGBoost, elastic net, and specialized single-cell panel-selection approaches, can capture predictive patterns but typically lack explicit spatial modeling and probabilistic feature selection, relying instead on model-specific importance scores or user-specified panel sizes. We develop rapid kernel machine regression (RKMR), a scalable framework for spatial-omics marker discovery that integrates nonlinear kernel modeling, spike-and-slab variable selection, and spatial dependence. RKMR uses automatic relevance determination (ARD) kernels and sparsity-inducing priors to capture nonlinear marker–outcome relationships and implicit feature interactions while producing approximate posterior inclusion probabilities (PIPs) that quantify model-based uncertainty in feature inclusion. To scale inference to large spatial datasets, RKMR combines low-rank kernel approximations with stochastic variational optimization. In simulations, RKMR consistently achieves higher AUPRC than competing methods across a range of molecular-signal and spatial-effect settings. Across spatial transcriptomics and scRNA-seq datasets, RKMR identifies parsimonious marker sets that recover reported cell-type signatures and reproducible tissue-layer markers. These results establish RKMR as a scalable and uncertainty-aware framework for translating high-dimensional spatial omics data into robust, experimentally actionable marker panels.

## Full Text Availability

The license terms selected by the author(s) for this preprint version do not permit archiving in PMC. The full text is available from the preprint server.

